# The use of urinary kidney injury molecule-1 and neutrophil gelatinase-associated lipocalin for diagnosis of hepato-renal syndrome in advanced cirrhotic patients

**DOI:** 10.1080/0886022X.2024.2346284

**Published:** 2024-05-17

**Authors:** Mohamed Adel Abd Elaziz, Asmaa Mustafa Gouda Elewa, Dina Zaki Mohamed Zaki Abdel Hamid, Nohier Essam Soliman Ahmed Hassan, Éva Csongrádi, Emad Hamdy Hamouda Mohammed, Mohammed Abdel Gawad

**Affiliations:** a Tropical Medicine Department, Faculty of Medicine, Alexandria University, Alexandria, Egypt; b Department of Clinical and Chemical Pathology, Faculty of Medicine, Alexandria University, Alexandria, Egypt; c Clinical Pharmacist at Alexandria Hepatology, Gastroenterology and Fever Hospital, Egypt; d Clinical Pharmacist at Zamzam Hospital, Alexandria, Egypt; e Faculty of Medicine, University of Debrecen, Debrecen, Hungary; f Lecturer of Critical Care, Faculty of Medicine, Alexandria University, Alexandria, Egypt; g Nephrology Unit, Internal Medicine Department, School of Medicine, Newgiza (NGU) University, Giza, Egypt

**Keywords:** Hepatorenal syndrome, neutrophil gelatinase-associated lipocalin, urinary kidney injury molecule-1, serum creatinine, liver cirrhosis

## Abstract

**Background:**

Chronic liver disease is a common and important clinical problem.Hepatorenal syndrome (HRS) is a life threatening complication. Serum creatinine (Cr) remains the only conventional indicator of renal function. However, the interpretation of serum Cr level can be confounded by malnutrition and reduced muscle mass often observed in patients with severe liver disease. Here, we present a cross-sectional study to explore the sensitivity and specificity of other markers as urinary KIM-1 and NGAL for cases of HRS.

**Methods:**

Cross-sectional study was conducted on 88 patients who were admitted to Alexandria main university hospital. Enrolled patients were divided in two groups; group 1: patients with advanced liver cirrhosis (child B and C) who have normal kidney functions while group 2: patients who developed HRS. Stata© version 14.2 software package was used for analysis.

**Results:**

Group 1 included 18 males and 26 females compared to 25 males and 19 females in group 2 (*p* = 0.135). Only the urinary KIM-1 showed a statistically significant difference between both groups in the multivariate logistic regression analysis adjusted for gender, serum bilirubin, serum albumin, INR, serum K, AST and ALT levels.

**Conclusion:**

In conclusion, our study aligns with prior research, as seen in the consistent findings regarding Urinary NGAL elevation in cirrhotic patients with AKI. Urinary KIM-1, independent of Urinary NGAL, may have a role in precisely distinguishing between advanced liver cirrhosis and HRS and merits further exploration.

## Introduction

Chronic liver disease is a common and severe clinical problem [1]. Hepatorenal syndrome (HRS) is a life-threatening complication with an incidence rate of 20% at 12 months in patients with advanced cirrhosis [2]. Serum creatinine (Cr) remains the only conventional indicator of renal function. However, the interpretation of serum Cr level can be confounded by malnutrition and reduced muscle mass often observed in patients with severe liver disease [3].

More importantly, serum Cr abnormality occurs late, only when there is already a significant (∼50%) reduction in glomerular filtration rate (GFR). Relying on serum Cr alone or Cr-based equations results in delayed diagnosis and HRS management. Biomarkers that could identify patients at an increased risk of developing HRS are thus important. Various serum and urinary biomarkers of acute kidney injury (AKI) have been proposed to supplement serum Cr in detecting impending or early renal impairment. These include serum Cystatin C (CysC), serum and urine neutrophil gelatinase-associated lipocalin (NGAL), serum interleukin-18 (IL-18), serum NAcetyl-β-D glucosaminidase (NAG) as well as the urinary kidney injury molecule-1 (KIM-1) and the liver-type fatty acid binding protein (LFABP) [4,5]. The aim of this work is to explore the sensitivity and specificity of urinary KIM-1 and NGAL for cases of hepatorenal syndrome.

## Materials and methods

This cross-sectional study was conducted on 88 patients with advanced liver cirrhosis (Child’s B or C cirrhosis) who were admitted to a tertiary university hospital between the 15th of June, 2023 and the 1^st^ of November, 2023. After the Institutional Review Board in the Faculty of Medicine, Alexandria University Ethics Committee approved the study protocol (Approval code: 0306200), all enrolled patients signed an informed consent. Enrolled patients were divided into two groups; Group 1 included patients with advanced liver cirrhosis (child B and C), regardless of its etiology. These patients had normal baseline kidney functions and were admitted for any reason other than renal impairment such as hepatic encephalopathy or jaundice, while group 2 included patients who developed HRS according to the criteria set by the European Association of Liver Study (EASL): 1. cirrhosis with ascites; 2. serum creatinine greater than 1.5 mg/dL; 3. absence of shock; 4. absence of hypovolemia as defined by non-sustained improvement of renal function following at least 2 days of diuretic cessation and volume expansion with a 1 g/kg/day albumin (maximum of 100 g/day); 5. No current or recent treatment with nephrotoxic drugs. All enrolled HRS patients were type-1 HRS AKI. Both group 1 and group 2 received liver support medications and anti-hepatic encephalopathy measures. Exclusion criteria included patients younger than 18 years of age, patients known to have chronic kidney disease (CKD), and patients with a recent history of nephrotoxic medications. Demographic data and etiology of liver cirrhosis were documented. Complete blood picture (CBC), coagulation profile, liver and kidney function tests, and spot urine samples (for measurement of urinary NGAL, and KIM-1) were collected between the 15^th^ of June and the 1^st^ of November, 2023. The estimated glomerular filtration rate (eGFR) was calculated using the CKD-EPI equation.

### Statistical analysis

The Stata© version 14.2 software package was used for analysis. Continuous variables were tested for normality using the Shapiro–Walk test. Normally distributed variables were expressed as mean (standard deviation) and were compared using the ANOVA test between the studied groups. Non-normally distributed variables were expressed as median and were compared using the Kruskal–Wallis test between the studied groups. Categorical variables were expressed as numbers and compared as percentages using the Chi-square test. Univariate logistic regression analysis was done to express the association between urinary biomarkers (KIM-1 and NGAL) and the presence of hepatorenal syndrome. Then a multivariate logistic regression model was adjusted for gender, bilirubin, albumin, INR, K, AST, and ALT. We took a cutoff value for urinary KIM-1 at 2.40 (the 75% quartile) and determined the area under curve (AUC), sensitivity, specificity, positive predictive value, and negative predictive value based on this cutoff.

## Results

This study included 88 patients admitted to Alexandria Main University Hospital. Group 1 included 18 males and 26 females, while there were 25 males and 19 females (*p* = 0.135) in group 2. The mean age was 59.3 (± 10.1) and 61.6 (± 9.9) years in the two groups, respectively (*p* = 0.293). Mean levels of kidney function tests, including serum Cr and blood urea, showed significantly higher levels in group 2 (*p* = 0.001), while eGFR was significantly lower in group 2 (*p* = 0.001). Median baseline serum potassium levels were 3.5 and 5.3 mEq/dL in both groups, respectively (*p* = 0.001). The median level of serum aspartate transaminase (AST) and the mean level of alanine transaminase (ALT) were also elevated significantly in group 2 (*p* = 0.001). Group 2 also showed a significant rise in their median INR (*p* = 0.009). Table 1 shows the baseline characteristics of the two groups.

**Table 1.** Baseline characteristics of the studied groups.

	All patients (*n* = 88)	Group 1 Patients without HRS (*n* = 44)	Group 2 Patients with HRS (*n* = 44)	*p* Value
Sex M/F	43/ 45	18/ 26	25/ 19	0.135
Age (years)	60.4 (9.9)	59.3 (10.1)	61.6 (9.9)	0.293
Weight (Kg)	78.2 (11.5)	76.5 (11.2)	79.8 (11.7)	0.176
Baseline Heart Rate (/ min)*	72	77	71	0.342
Baseline WBCs (cells/ mm^3^)	5100	4690	5650	0.473
Baseline s. creatinine (mg/ dL)*	1.36	0.9	2.85	0.001**
Baseline blood urea (mg/ dL)*	70	50	99.5	0.001**
eGFR (mL/min)*	49.9	82.5	23.1	0.001**
Baseline Na (mEq/L)	130.0 (5.2)	130.5 (5.7)	129.6 (4.6)	0.445
Baseline K (mEq/L)*	4	3.5	5.3	0.001**
Baseline AST (Units/L)* (normal range is 8 to 33 Units/L)	65.5	50.5	85	0.001**
Baseline ALT (Units/L) (normal range is 4 to 36 Units/L)	45.3 (16.6)	38.1 (9.8)	52.4 (18.9)	0.001**
Baseline total bilirubin (mg/ dL)*	2.1	1.9	2.1	0.399
Baseline s. albumin (g/dL)	2.9 (0.6)	3.1 (0.7)	2.8 (0.6)	0.062
Baseline INR*	1.6	1.5	1.7	0.009**
Etiology of liver cirrhosis:				0.336
HCV	82	42	40	
Bilharzial	2	0	2	
Budd Chiari	1	1	0	
Unidentified	3	1	2	
Ascites by ultrasound:				0.816
Nil	7	4	3***	
Mild	30	15	15	
Moderate	38	20	18	
Tense	13	5	8	
Spleen by ultrasound:				0.952
Normal	30	15	15	
Splenectomy	13	6	7	
Splenomegaly	45	23	22	

n: number; P: probability value; HRS: hepatorenal syndrome; M/F: male/female; KG: kilogram; min: minute; cells/mm^3^: cells per cubic millimeter; mg/dL: milligrams per deciliter; eGFR: estimated glomerular filtration rate; mL/min: milliliter per minute; Na: sodium; mEq/L: milliequivalents per liter; K: potassium; AST: aspartate aminotransferase; Units/L: units/Liter; ALT: alanine transaminase; s: albumin = serum bilirubin; g/dL: gram per deciliter; INR: international normalized ratio; HCV: hepatitis C.

* : not normally distributed variables,.

** : statistically significant difference.

*** These cases presented clinically with ascites, but they had ultrasound done late after repeated paracentesis as they presented with respiratory distress and were in need for urgent paracentesis. Paracentesis was done blindly without imaging guidance.

The median level of urinary KIM-1 was 0.89 and 1.4 ng/mL in group 1 and group 2, respectively (*p* = 0.282). As for urinary NGAL, the median level was 1.53 in group 1 and 1.77 ng/mL in group 2 (*p* = 0.104) (Table 2).

**Table 2.** . Different levels of U-NGAL and U-KIM-1 in groups 1 and 2.

	All patients (*N* = 88)	Group 1 Patients without HRS (*n* = 44)	Group 2 Patients with HRS (*n* = 44)	*p* Value
Urinary KIM-1(ng/mL)*	0.03 − 3.39 (0.61)	0.48 − 3.38 (0.89)	0.03 − 3.39 (1.4)	0.282
Min – max (median)				
Urinary N-GAL (ng/mL)* Min – max (median)	0.04 − 2.70 (1.63)	0.45 − 2.37 (1.53)	0.04 − 2.70 (1.77)	0.104

* not normally distributed variables.

**Figure 1.**
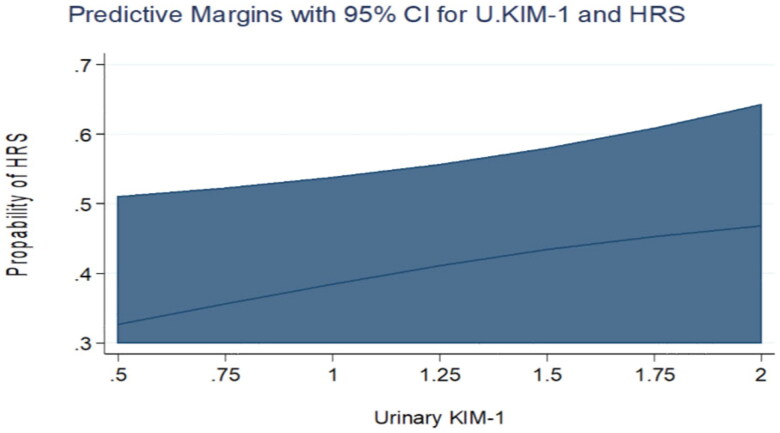
Predictive margins for Urinary KIM-1 and HRS with 95% confidence intervals. The increasing levels of Urinary KIM-1 were not associated with an increasing probability of HRS in the univariate analysis.

**Figure 2.**
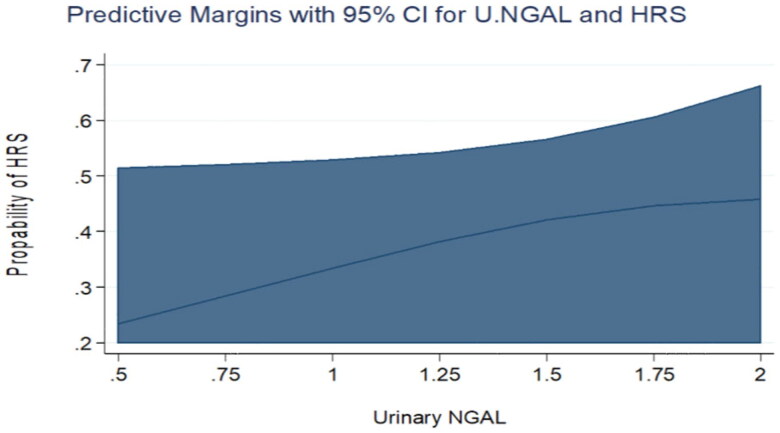
Predictive margins for Urinary NGAL and HRS with 95% confidence intervals. The increasing levels of Urinary NGAL were not associated with an increasing probability of HRS in the univariate analysis.

**Figure 3.**
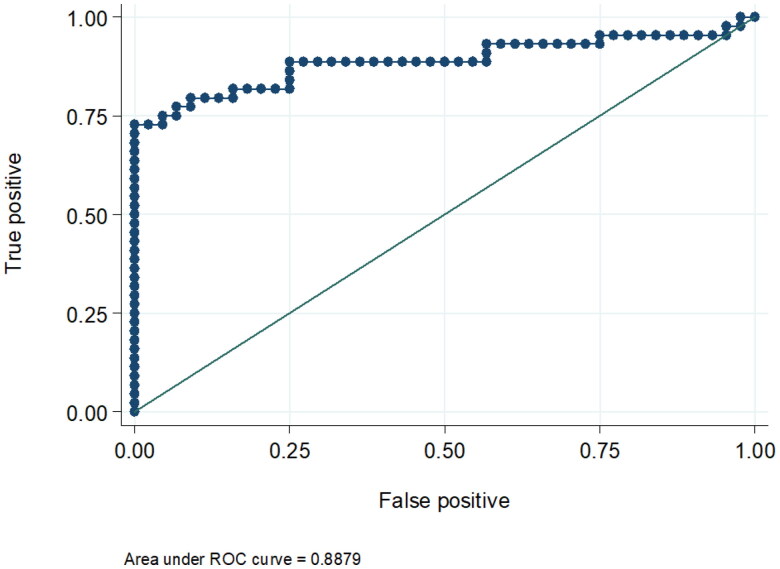
shows the receiver operating curve (ROC) for the sensitivity and specificity of Urinary KIM-1 for the prediction of the occurrence of HRS. At the level of 2.4 ng/mL (the 75th percentile), the area under curve was 0.88. Specificity and sensitivity were 61.1% and 52.9%, respectively at the same level.

Comparing the two groups, urinary KIM-1 and urinary NGAL levels were comparable in both groups by univariate logistic regression analysis. The odds ratio for elevated urinary KIM-1 was 1.42 (95% CI was 0.923 − 2.19, *p* = 0.110) and 1.73 for elevated urinary NGAL (95% CI was 0.817 − 3.686, *p* = 0.152). Only urinary KIM-1 showed a statistically significant difference between the two groups in the multivariate logistic regression analysis (OR= 2.2, 95% CI was 1.10- 4.415, *p* = 0.026) adjusted for gender, serum bilirubin, serum albumin, INR, serum K, AST and ALT levels. (Table 3, Figures 1,2). The receiver operating curve (ROC) for the sensitivity and specificity of urinary KIM-1 for the prediction of occurrence of HRS showed that at the level of 2.4 ng/mL (the 75th percentile), the area under curve was 0.88. Specificity and sensitivity were 61.1% and 52.9%, respectively at the same level. (Table 4, Figure 3).

**Table 3.** Association between urinary KIM-1 and NGAL and the presence of HRS.

	Univariate analysis			Multivariate analysis		
	OR	95% CI	*p*	OR	95% CI	*p*
Urinary KIM-1	1.422	0.923 − 2.191	0.110	2.204	1.101 − 4.415	0.026**
Urinary NGAL	1.735	0.817 − 3.686	0.152	2.913	0.838 − 10.124	0.093

Multivariate analysis adjusted for gender, bilirubin, albumin, INR, K, AST, ALT.

**Table 4.** Estimation of the cutoff value of U-KIM-1 in patients with HRS.

	Cutoff value	AUC	Sensitivity	Specificity	Positive predictive value	Negative predictive value
Urinary KIM-1	2.40	0.888	61.1 %	52.9 %	25 %	84.1 %

## Discussion

AKI occurs in 20–50% of hospitalized patients with advanced cirrhosis, leading to high mortality rates [6]. AKI in patients with liver cirrhosis has a wide range of differential diagnoses. Recognizing the AKI phenotype early is crucial since treatment approaches vary depending on the cause, which can have an impact on recovery. This study highlights the possible role of urinary KIM-1 in identifying patients with HRS, a condition with a challenging diagnosis and a poor outcome, with an evident need for an accurate biomarker for precise diagnosis and early intervention.

While the baseline characteristics of the two groups were not matched, this is fairly understandable. Kidney function tests and serum potassium were, as expected, worse in the HRS group compared to the non-HRS group. Similarly, liver function tests were worse in the HRS group. This was shown also in a previous study from Egypt, where the HRS group showed elevated bilirubin, INR, MELD scores, lower serum albumin, and more attacks of hepatic encephalopathy and spontaneous bacterial peritonitis in comparison to patients with cirrhosis without renal impairment and patients with cirrhosis without ascites [7]. In our study, ALT and AST were significantly higher in the HRS group. This may indicate that patients with a worse liver profile are more susceptible to the development of HRS than those with a better liver profile.

KIM-1 is a 38.7-kDa type I transmembrane glycoprotein expressed at low levels in the kidney and other tissues, but upon kidney injury, it is markedly increased [8]. In our study, as shown by the multivariate logistic regression analysis after adjustment for gender, serum bilirubin, serum albumin, INR, serum potassium, AST and ALT levels, urinary KIM-1 was significantly higher in the HRS group. Studies on urinary KIM-1 in hepatorenal syndrome are scarce and show contradictory results. In 2014, a study used urinary KIM-1 in patients with cirrhosis to identify the cause of AKI. 53% of patients were diagnosed with acute tubular necrosis (ATN), 26% with pre-penal azotemia and 22% with HRS. Urinary KIM-1 did not differ significantly in HRS compared to other causes of AKI [4]. Another study showed that urinary KIM-1 level elevations in cirrhosis patients were primarily observed in ATN compared to other AKI presentations [9]. On the other hand, another study showed that urinary KIM-1 was significantly higher in the HRS group and could predict HRS-AKI at a cutoff point of > 3.1 pg/ml [7]. These conflicting results may result from the complex nature of the disease, the presence of multiple comorbidities and the small sample size of all published studies.

NGAL is a protein excreted in urine by renal tubules. It has been used as a marker of tubular injury. Higher urinary NGAL is associated with acute tubular damage, therefore it could differentiate the cause of kidney impairment in patients with liver cirrhosis [4,10]. Our study did not show a significant difference in urinary NGAL levels between patients with liver cirrhosis who had normal kidney functions and those who developed HRS. Many studies found significantly higher levels of urinary NGAL in patients with ATN compared to HRS, and higher levels of NGAL in HRS compared to pre-renal AKI [9,11–14]. On the other hand, two studies found no difference in urinary NGAL between HRS and pre-renal AKI and no significant change in urinary NGAL levels at any time point between patients with resolved HRS and patients who did not respond [15,16]. Nevertheless, Gambino et al. reported that high urinary NGAL levels were associated with non-response to terlipressin plus albumin therapy [17].

In summary, the reliability of NGAL in HRS is still questionable. Most of the published studies are on small cohorts and the diagnosis of ATN and pre-renal AKI is based on investigations other than kidney biopsy. Moreover, NGAL is secreted from other organs, such as the gastrointestinal tract and the lung and could be increased in different clinical settings, such as infection, sepsis and surgery [18,19].

The limitations of our study include a small number of participants, being a single center study, a dependence on a single measurement of biomarkers rather than sequential measurements and the absence of standard time for measurement after developing kidney dysfunction.

## Conclusion

In conclusion, our study aligns with prior research, as seen in the consistent findings regarding urinary NGAL elevation in patients with cirrhosis and AKI. Urinary KIM-1, independent of urinary NGAL, may have a role in precisely distinguishing between advanced liver cirrhosis and HRS and merits further exploration.

## References

[1] Su Y, Yan R, Duan Z, et al. Prevalence and risk factors of hepatitis C and B virus infections in hemodialysis patients and their spouses: a multicenter study in Beijing, China. J Med Virol. 2013;85(3):1–6. doi: 10.1002/jmv.23486. 23341370

[2] Seo YS, Jung ES, An H, et al. Serum cystatin C level is a good prognostic marker in patients with cirrhotic ascites and normal serum creatinine levels. Liver Int. 2009;29(10):1521–1527. doi: 10.1111/j.1478-3231.2009.02105.x. 19725889

[3] Cholongitas E, Shusang V, Marelli L, et al. Review article: renal function assessment in cirrhosis - difficulties and alternative measurements. Aliment Pharmacol Ther. 2007;26(7):969–978. doi: 10.1111/j.1365-2036.2007.03443.x. 17877504

[4] Belcher JM, Sanyal AJ, Peixoto AJ, et al. Kidney biomarkers and differential diagnosis of patients with cirrhosis and acute kidney injury. Hepatology. 2014;60(2):622–632. doi: 10.1002/hep.26980. 24375576 PMC4065642

[5] Yap DY, Seto WK, Fung J, et al. Serum and urinary biomarkers that predict hepatorenal syndrome in patients with advanced cirrhosis. Dig Liver Dis. 2017;49(2):202–206. doi: 10.1016/j.dld.2016.11.001. 27876501

[6] Tandon P, James MT, Abraldes JG, et al. Relevance of new definitions to incidence and prognosis of acute kidney injury in hospitalized patients with cirrhosis: a retrospective population-based cohort study. PLoS One. 2016;11(8):e0160394. doi: 10.1371/journal.pone.0160394. 27504876 PMC4978466

[7] El-Makarem M-RA, et al. Do old urinary biomarkers have a place in the new definition of hepatorenal syndrome in the Egyptian cirrhotic patients? A single-center experience. Egypt Liver Journal*.* 2022;12(1):23.

[8] Geng J, et al. The value of kidney injury molecule 1 in predicting acute kidney injury in adult patients: a systematic review and Bayesian meta-analysis. J Transl Med*.* 2021;19(1):1–13. 10.1186/s12967-021-02776-8 PMC7953563 33712052

[9] Qasem AA, Farag SE, Hamed E, et al. Urinary biomarkers of acute kidney injury in patients with liver cirrhosis. ISRN Nephrology. 2014;2014:1–7. doi: 10.1155/2014/376795. PMC4045442 24967242

[10] Allegretti ASJH. NGAL in AKI and cirrhosis—ready for prime time? Hepatology. 2023;77(5):1472–1474. doi: 10.1002/hep.32807. 36169601 PMC10043041

[11] Fagundes C, Pépin M-N, Guevara M, et al. Urinary neutrophil gelatinase-associated lipocalin as biomarker in the differential diagnosis of impairment of kidney function in cirrhosis. J Hepatol. 2012;57(2):267–273. doi: 10.1016/j.jhep.2012.03.015. 22521351

[12] Verna EC, Brown RS, Farrand E, et al. Urinary neutrophil gelatinase-associated lipocalin predicts mortality and identifies acute kidney injury in cirrhosis. Dig Dis Sci. 2012;57(9):2362–2370. doi: 10.1007/s10620-012-2180-x. 22562534 PMC3979299

[13] Ariza X, Solà E, Elia C, et al. Analysis of a urinary biomarker panel for clinical outcomes assessment in cirrhosis. PLoS One. 2015;10(6):e0128145. doi: 10.1371/journal.pone.0128145. 26042740 PMC4456079

[14] Jaques DA, Spahr L, Berra G, et al. Biomarkers for acute kidney injury in decompensated cirrhosis: a prospective study. Nephrology (Carlton). 2019;24(2):170–180. doi: 10.1111/nep.13226. 29369449

[15] Huelin P, Solà E, Elia C, et al. Neutrophil gelatinase-associated lipocalin for assessment of acute kidney injury in cirrhosis: a prospective study. Hepatology. 2019;70(1):319–333. doi: 10.1002/hep.30592. 30810244

[16] Solé C, Ma AT, Solà E, et al. Sequential changes in urinary biomarker levels in patients with cirrhosis and severe hepatorenal syndrome. Liver Int. 2021;41(11):2729–2732. doi: 10.1111/liv.15069. 34569697

[17] Gambino C, et al. Diagnostic and prognostic performance of urinary neutrophil gelatinase-associated lipocalin in patients with cirrhosis and acute kidney injury. Hepatology. 2023;77(5):1630–1638. 10.1002/hep.32799 PMC10113003 36125403

[18] Cowland JB, Borregaard NJG. Molecular characterization and pattern of tissue expression of the gene for neutrophil gelatinase-associated lipocalin from humans. Genomics. 1997;45(1):17–23. doi: 10.1006/geno.1997.4896. 9339356

[19] Manfredi MA, Zurakowski D, Rufo PA, et al. Increased incidence of urinary matrix metalloproteinases as predictors of disease in pediatric patients with inflammatory bowel disease. Inflamm Bowel Dis. 2008;14(8):1091–1096. doi: 10.1002/ibd.20419. 18338781

